# Risk factors and short and medium-term survival after open and endovascular repair of abdominal aortic aneurysms

**DOI:** 10.1590/1677-5449.011717

**Published:** 2018

**Authors:** Seleno Glauber de Jesus-Silva, Victor Rodrigues de Oliveira, Melissa Andreia de Moraes-Silva, Arturo Eduardo Krupa, Rodolfo Souza Cardoso

**Affiliations:** 1 Hospital de Clínicas de Itajubá – HC, Serviço de Cirurgia Vascular e Endovascular, Itajubá, MG, Brasil.; 2 Hospital de Clínicas de Itajubá – HC, Serviço de Cirurgia Geral, Itajubá, MG, Brasil.

**Keywords:** abdominal aortic aneurysm, risk factors, blood vessel prosthesis implantation, survival analysis

## Abstract

**Background:**

Infrarenal abdominal aortic aneurysms (AAA) are responsible for high rates of rupture-associated morbidity and mortality and can be treated by open or endovascular surgery.

**Objectives:**

To analyze risk factors and survival associated with surgical and endovascular AAA treatment methods.

**Methods:**

A retrospective, longitudinal study involving 41 patients who underwent endovascular or open AAA repair, whether elective or emergency, over a 48-month period, with analysis of preoperative comorbidities, 30-day and 1-year survival, in-hospital mortality, length of hospital stay, transfusion of blood products, duration of surgery, and development of acute kidney failure. Inferential statistics and survival analysis considered a 95% CI and p < 0.05 as significant.

**Results:**

Twelve of the 41 patients were treated with open surgery and 29 with endovascular techniques. The majority were male (75%), with an average age of 71 (range: 56 – 90 years). There were no differences in demographic or risk factors between the groups. Overall survival rates for open and endovascular repair were different for both 30 days (37 vs. 72%, p = 0.01) and 360 days (37 vs. 67%, p = 0.01). However, survival rates in elective cases were similar at 30 days (71 vs. 76%, p = 0.44) and 360 days (both 71%, p = 0.34). Endovascular repair showed shorter length of hospital stay (3.0 vs. 4.4 days; p = 0.02) and duration of surgery (111 vs. 163 min; p < 0.01) compared to open repair.

**Conclusions:**

There was no difference in short- or medium-term survival of AAA patients treated electively with endovascular or open surgery. Hospital stays and duration of surgery were both shorter with minimally invasive treatment.

## Introduction

 Infrarenal abdominal aortic aneurysms (AAA) are the most common type and occur in around 2.3% of the general population, 1 and as much as 5.96% of men over the age of 60 years. Furthermore, there is a possibility of complications including rupture, when mortality can be as high as 80 to 90%. 2 Some risk factors associated with development of AAA are well-defined, such as advanced age, male sex, smoking, family history, and presence of other aneurysms in large vessels. 3 ^,^ 4

 Open surgical repair is considered effective and definitive and has been performed since 1951. However, this technique is associated with non-negligible morbidity and mortality rates, long periods in hospital, and a need for blood transfusion. Mortality rates associated with elective surgery can range from 5 to 10%. 5 ^,^ 6 Endovascular treatments have been in development since 1991 as an alternative option for high-risk patients who cannot be subjected to open surgery. Nowadays, with the accumulation of experience and development of safer and more flexible prostheses, endovascular treatment can be considered the method of choice, even for patients whose surgical risk assessments and anatomic characteristics are favorable for the conventional open surgical technique. 6 ^,^ 7 Controlled trials and cohort studies have shown lower short-term perioperative morbidity and mortality with endovascular repair than with open surgical repair. However, the long-term survival curves for the two techniques are similar. The incidence of reinterventions is also higher after endovascular repair than after open surgical repair. 6 ^-^ 11

 In view of the scarcity of published data from Brazil on the comparative outcomes of the two techniques used to manage AAA, the objective of this study is to analyze the main risk factors and the short (up to 30 days) and medium-term (up to 1 year) survival of patients treated with open and endovascular repair in a quaternary hospital. The study was approved by the institution’s Research Ethics Committee under protocol number 2.069.326. 

## Methods

 This is a retrospective study conducted by analysis of the medical records of 45 patients treated with open or endovascular repair of infrarenal AAA from March 2013 to March 2017 in a quaternary hospital. There was no formal randomization of the patients treated at this service to choose the method employed (open or endovascular repair). However, the decision of which technique to use was taken in team meetings after analysis of tomographic anatomy, comorbidities, and surgical risk assessments. Elective patients with favorable anatomy (proximal neck > 25 mm in length or angle < 60º and external iliac arteries with diameter > 7 mm) or those with borderline anatomy (proximal neck from 15 to 25 mm in length or angle from 60º to 70º), but with a high surgical risk, were treated using the minimally invasive technique. The remaining elective cases were treated with open surgery. For urgent cases (ruptured or acutely expanding aneurysms), the technique was chosen based on stability of clinical status, favorability of anatomy, and immediate availability of endoprostheses. Each patient studied only underwent one aneurysm repair procedure. Data on a total of 10 preoperative clinical variables and eight postoperative clinical variables were collected and input to an electronic spreadsheet. Four medical records for surgical patients were incomplete (two did not contain data on duration of surgery, one did not have complete laboratory test results, and one did not have an accessible imaging exam that could be used to analyze aneurysm diameter) and were excluded from the study, leaving a total of 41 medical records for analysis. Correlations between the anatomic characteristics of the aneurysms and their outcomes were not studied because there was incomplete availability of examinations that could be used for reconstruction. 

 Systemic arterial hypertension was defined as pressure greater than 140 x 90 mmHg or continuous use of antihypertensive; diabetes mellitus as fasting glycemia > 106 mg/dL or use of hypoglycemics; smoking as prior or current use of tobacco or derivatives; kidney failure as creatinine clearance < 60 mL/min or serum creatinine > 1.5 mg/dL; and peripheral arterial occlusive disease as an ankle-brachial index < 0.9 or evident clinical signs of arterial occlusion. Other parameters analyzed were history of acute myocardial infarction less than 6 months previously, stroke, angina, abdominal pains, and aneurysm diameter. Ruptured AAA were diagnosed with imaging exams (ultrasound or computed tomography). The data collected for variables after AAA repair were hospital mortality (occurring during the surgical procedure or in the immediate postoperative period), overall mortality (death from any cause, outside of the hospital setting, after discharge), time in an intensive care unit for less than 24 hours, need for blood transfusion intraoperatively or postoperatively, acute kidney failure (increase of 0.5 mg/dL or increase of 25% over baseline), length of hospital stay after AAA repair, and duration of surgery. The follow-up period chosen for survival analysis was up to 360 days. 

 The descriptive statistics calculated were means and standard deviations. Intergroup inferential analysis (open surgery vs. endovascular repair) was conducted using Student’s *t* test for independent samples, the Mann-Whitney test or Fisher’s exact test. Survival was analyzed using Kaplan-Meier curves with the log-rank test for comparison between groups. Graphpad Prism version 7.0c was used, with 95% confidence interval (CI) and statistical significance to p < 0.05. 

## Results

 Twenty-nine of the 41 patients treated for AAA underwent endovascular repair and 12 underwent open surgery. The majority were male (n = 29; 70.7%) and the mean age of the patients was 71 (range 56-90 years). Fourteen patients died during the study period. Nine cases (22%) involved ruptured AAA, among whom two out of four patients treated with endovascular procedures survived, while four out of five patients treated with open surgery died in the immediate postoperative period. Up to the end of the period studied, just one of the open surgery cases and nine endovascular treatment cases were still in outpatient follow-up. 

Table 1 lists the risk factors for both groups of patients. No statistical differences between the groups were detected. Table 2 shows the comparison between the postoperative characteristics of the groups of patients treated with open and endovascular surgery. It was observed that overall hospital mortality, length of hospital stay, and duration of surgery were all statistically lower in the endovascular treatment group. The length of hospital stay analysis only included patients who were actually discharged from hospital, with a median of 4 days for open surgery (range: 3-6 days) and 2 days for endovascular treatment (range: 1-10 days). 

**Table 1:** Preoperative risk factors observed in open surgery and endovascular repair groups.

**Risk factors**	**Open**		**Endovascular**		**p**
	**n**	**%**	**n**	**%**	
Sex					
Male	8	67	23	79	0.44
Female	4	33	6	21	
Age (years)	69	7.2 (SD)	72	9.5 (SD)	0.32
SAH	10	83	23	79	0.99
Diabetes mellitus	1	8.3	6	20.7	0.65
Smoking	10	83	6	55	0.15
CKF	1	8.3	7	24	0.40
AMI < 6 m	0	0	2	8.3	0.54
Angina	2	17	2	6.9	0.56
Stroke	0	0	1	3.6	0.99
PAOD	1	8.3	5	18	0.64
Abdominal pain	7	58	13	44	0.50
Ruptured AAA	5	42	4	14	0.09
Aneurysm diameter (cm)	6.8	2.3 (SD)	6.1	1.7 (SD)	0.30

AAA, abdominal aortic aneurysm; PAOD, peripheral arterial occlusive disease; SD, standard deviation; SAH, systemic arterial hypertension; AMI, acute myocardial infarction; CKF, chronic kidney failure.

**Table 2:** Comparison of outcome variables in treatment groups (open surgery and endovascular repair).

**Outcomes**	**Open**		**Endovascular**		**p**
	**n**	**%**	**n**	**%**	
Hospital mortality	7	58	4	14	0.006
Overall mortality (1 year) ^*^	7	58	7	24	0.06
Hospital mortality (elective patients)	2	29	2	8	0.18
ICU < 24 h	1	6.7	5	31	0.17
Blood transfusion	6	60	10	34	0.26
Post-procedure AKF	2	20	6	21	0.99
Length of hospital stay (days) ^*^	4.4	1.1 (SD)	3.0	1.9 (SD)	0.02
Duration of surgery (min)	163	36 (SD)	115	46 (SD)	0.005

SD, standard deviation; AKF, acute kidney failure; ICU, intensive care unit;

* Excluding in-hospital deaths.

**Figure 1:**
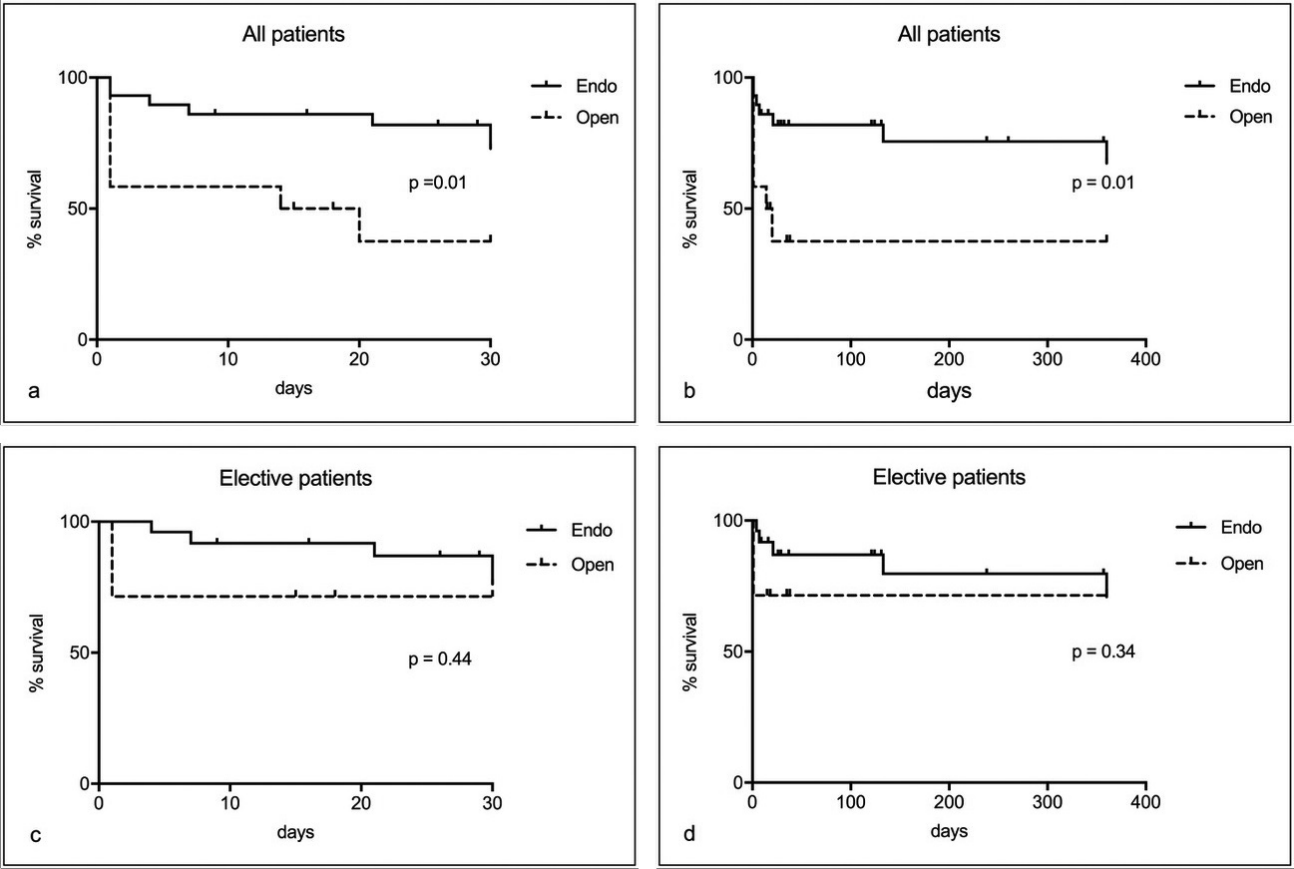
Kaplan-Meier survival curves with log-rank test for patients treated. In a and b, the global survival curves for all patients (elective and emergency) for 30 and 360 days show significant differences between groups. In c and d, showing an analysis of elective patients only, there is no difference in survival related to the treatment technique chosen.

 Survival was analyzed both for the entire sample (combining urgent and elective cases) and for elective cases only, for short (up to 30 days) and medium term (up to 1 year), and for both types of treatment, open and endovascular ( Figure 1 ). A significant difference in global survival was observed for patients treated with endovascular techniques, irrespective of follow-up period. However, when only the elective cases were analyzed there was no difference in short or medium-term survival. Thirty-day survival among elective cases was 71% after open surgery and 76% after endovascular repair (p = 0.44), and at 360 days the rates were both 71% (p = 0.34). In the analysis of all patients treated, 30-day survival was 37% for open repair and 72% for endovascular repair (p = 0.01) and 360-day survival rates were 37% and 67%, respectively (p = 0.01). 

## Discussion

 Whereas open AAA repair was first achieved in 1951 by Dubost and has remained a standard treatment ever since, endovascular repair was not conducted successfully until 1990, by Parodi et al. 12 Since then it has become an alternative option to open surgery. The endovascular procedure was developed with the objective of offering a less traumatic treatment for aneurysm repair, as an option for use in the elderly, high risk patients, and those with concurrent diseases that impact on the risk of the conventional procedure. It has a high success rate and fewer perioperative complications than open surgical repair. Complications are generally related to technical issues, such as difficulty with vascular access or with placement of the prosthesis, structural integrity, migration of the prosthesis, and endoleaks. However, conversion to open repair is rare and when late complications occur they can also be treated with endovascular techniques. 13 ^,^ 14 Currently, the preferred indication is the minimally invasive technique and open surgery is reserved for those patients in whom the anatomic conditions for implantation of an endoprosthesis are not present or who have an unstable, ruptured AAA. This is corroborated by our findings showing no statistical differences between the epidemiological characteristics of the two groups. 

 Even when treated with open surgical repair, patients with ruptured AAA have mortality of approximately 50%, a rate that has not changed over recent years. 15 Patients with AAA with maximum transverse diameters from 5.5 to 5.9 cm have an annual rupture rate of 9.4%, which can rise up to 32.5% if the aneurysm reaches 7 cm. 16 However, open surgical repair involves prolonged recovery times and non-negligible perioperative mortality rates, 17 with variable rates, for example, 3.1% in the United Kingdom. 18 Some Brazilian publications report similar results, with rates of 3.3 to 5.3%. 19 ^-^ 21 However, depending on the anatomic configuration of the aorta and the access arteries, in cases with a short proximal neck, tortuosity, and dilatation, or where iliac vessels are tortuous or narrow, open repair is necessary. 

 A retrospective Brazilian study of patients who received endovascular treatment from June 1996 to February 2004, based on analysis of a database from the European Collaborators on Stent-graft Techniques for Abdominal Aortic Aneurysm Repair (EUROSTAR) project, found that mean duration of the procedure was 137 minutes (25 to 287 min) and mean hospital stay was 6 days (0 to 163). 22 In the present study, the median length of hospital stay was shorter for endovascular treatment (2 days) than for open surgery (4 days) and both were shorter than the study just cited. This could be because of improved techniques and materials and consolidation of the surgical team’s organization. The mean duration of the endovascular procedure was also shorter (115 min), for similar reasons. In the more recent EVAR-1 study, 23 duration of endovascular treatment was also no longer than for open repair, in agreement with what was observed in our study. 

 After the EVAR-1 study was conducted, 23 reduced perioperative mortality was confirmed (4.7% for open repair vs. 1.7% for endovascular repair). Another controlled and randomized study reported a lower rate for the endovascular technique (1.2 vs. 4.6%). 24 In Brazil, Mendonça et al. 25 compared open treatment with endovascular repair for AAA with favorable anatomy. Mortality was 6.45% for open treatment and 5.55% for endovascular treatment, with no statistically significant difference. In the present study we also did not observe a difference in in-hospital mortality between open and endovascular elective cases (29 vs. 8%, respectively; p = 0.18) or any difference in 30-day mortality (71 vs. 76%; p = 0.44). 

 Goshima et al. 26 claim that the standard result for open repair should be 3.1% and in their study there was zero mortality with endovascular treatment. Nevertheless, when the same authors presented results for complex cases, they reported hospital mortality of 14.1%, similar to our study, which included patients with ruptured aneurysms. In the EVAR-2 study 27 ^,^ 28 patients who were not suitable for open repair were randomized to endovascular treatment or clinical follow-up and 30-day mortality in the operated group was 9% (5-15%; 95%CI). This reinforces the idea that more complex cases can have higher mortality rates even when treated with endovascular repair. In our study, we found a similar situation, with a high 30-day mortality rate, even among elective cases and those treated with endovascular methods. In addition to taking into account the fact that we treat patients with high surgical risk, referred from other towns and without adequate control of risk factors, the fact that this is a teaching hospital with a multidisciplinary learning curve for treatment of aortic disease also introduces a bias that should not be ignored. 

 The Dutch multicenter DREAM study 24 also showed a tendency for lower operative mortality (within 30 days) with endovascular treatment when compared with open surgery. However, in our study it was observed that patients treated electively in both groups did not exhibit differenced in survival at 30 days or at 360 days. The nonrandomized technique selection will have played a fundamental role in the similar survival rates in both groups. 

 Notwithstanding, 2 and 4-year follow-up results from the DREAM and the EVAR-1 studies showed similar long-term mortality in both groups. 27 ^,^ 29 In our study, there was a difference in overall survival between the two groups (ruptures + elective patients) during the first 30 days, but over the medium term the results equaled out, in common with the study mentioned above. 

 Points that could be considered negative in relation to this retrospective study include the small number of cases, the loss to follow-up of patients who live in other micro-regions and the missing information on risk factors in some of the medical records analyzed. To improve understanding of the subject covered in this study, it is necessary to conduct studies with larger numbers of patients, preferably with multicenter collaborations, defining prospective protocols and conducting long-term follow-up (5 years). 

## Conclusions

 We observed that patients who underwent elective endovascular treatment exhibited short and medium-term survival that was similar to those treated with elective open surgery. Length of hospital stay, duration of surgery, and in-hospital mortality were lower in the endovascular group. There were no differences in epidemiological characteristics or in the presence of risk factors between patients treated with the two types of techniques and the choice of treatment was based on anatomic criteria and the surgeon’s judgment. 
