# Supplementary material for: Risk factors and short and medium-term survival after open and endovascular repair of abdominal aortic aneurysms
Source: J Vasc Bras. 2018 Jul-Sep;17(3):201–7. doi: 10.1590/1677-5449.011717 (PMC6326136; doi:10.1590/1677-5449.011717)
Supplement: Translation

# Translation: Fatores de risco associados e sobrevida em curto e médio prazo de pacientes submetidos a correção aberta e endovascular de aneurisma de aorta abdominal

**Authors:** Seleno Glauber de Jesus-Silva, Victor Rodrigues de Oliveira, Melissa Andreia de Moraes-Silva, Arturo Eduardo Krupa, Rodolfo Souza Cardoso

**Affiliations:** 1 Hospital de Clínicas de Itajubá – HC, Serviço de Cirurgia Vascular e Endovascular, Itajubá, MG, Brasil.; 2 Hospital de Clínicas de Itajubá – HC, Serviço de Cirurgia Geral, Itajubá, MG, Brasil.

**Keywords:** aneurisma da aorta abdominal, fatores de risco, implante de prótese vascular, análise de sobrevida

## Abstract

**Contexto:**

Os aneurismas de aorta abdominal (AAA) infrarrenal apresentam alta morbimortalidade associada à ruptura e podem ser tratados por cirurgia aberta ou endovascular.

**Objetivos:**

Analisar os fatores de risco e a sobrevida associados aos métodos cirúrgico e endovascular no tratamento do AAA.

**Métodos:**

Estudo retrospectivo e longitudinal envolvendo 41 pacientes submetidos à correção endovascular ou aberta do AAA, de forma eletiva ou emergencial, no período de 48 meses. Foi realizada análise de comorbidades pré-operatórias, sobrevida em 30 dias e 1 ano, mortalidade hospitalar, tempo de internação, hemotransfusões, duração da cirurgia e ocorrência de insuficiência renal aguda. A estatística inferencial e a análise de sobrevida foram realizadas considerando intervalo de confiança de 95% e p < 0,05 como significante.

**Resultados:**

Dos 41 pacientes, 12 foram submetidos à correção aberta e 29, à endovascular. A maioria eram homens (75%), com média de idade de 71 anos (mín. 56, máx. 90 anos). Não houve diferenças de fatores de risco entre os grupos. A sobrevida global dos pacientes foi diferente para os tratamentos aberto e endovascular, tanto em 30 dias (37 *vs.* 72%; p = 0,01) quanto em 360 dias (37 *vs.* 67%; p = 0,01), respectivamente. A sobrevida dos casos eletivos em 30 dias (71 *vs.* 76%; p = 0,44) e 360 dias (ambas 71%; p = 0,34) foram semelhantes. O reparo endovascular apresentou menor tempo de internação (3,0 *vs.* 4,4 dias; p = 0,02) e duração da cirurgia (111 *vs.* 163 min; p = 0,005) quando comparado à cirurgia aberta.

**Conclusões:**

Não houve diferença na sobrevida em curto e médio prazo dos pacientes com AAA tratados de forma eletiva pelas técnicas endovascular e cirúrgica. Menor tempo de internação e duração da cirurgia foram observados no tratamento minimamente invasivo.

## INTRODUÇÃO

 Os aneurismas de aorta abdominal (AAA) infrarrenal são os mais comuns e ocorrem em cerca de 2,3% da população geral, 1 podendo chegar a 5,96% em homens acima de 60 anos. Além disso, há chance de complicação com ruptura, cuja mortalidade pode chegar de 80 a 90%. 2 Alguns fatores de risco associados ao desenvolvimento do AAA já são bem definidos, como idade avançada, sexo masculino, tabagismo, história familiar e presença de outros aneurismas em grandes vasos. 3 ^,^ 4

 A correção cirúrgica aberta é considerada efetiva e definitiva, sendo realizada desde 1951. No entanto, a técnica possui taxas de morbimortalidade não desprezíveis, com internação prolongada e necessidade de hemotransfusão. A mortalidade na cirurgia eletiva pode alcançar de 5 a 10%. 5 ^,^ 6 O tratamento endovascular desenvolveu-se a partir de 1991 como uma alternativa para os pacientes de alto risco que não poderiam se submeter à cirurgia aberta. Atualmente, com a experiência acumulada e o desenvolvimento de próteses mais seguras e flexíveis, o tratamento endovascular pode ser considerado como o de escolha, mesmo em pacientes de risco cirúrgico e características anatômicas favoráveis à técnica cirúrgica aberta convencional. 6 ^,^ 7 Ensaios controlados e estudos de coorte têm mostrado menor morbidade e mortalidade perioperatórias em curto prazo da correção endovascular em relação ao reparo cirúrgico aberto. Entretanto, a curva de sobrevida em longo prazo de ambas as técnicas tem se mostrado semelhante. Nota-se ainda maior incidência de reintervenções no reparo endovascular quando comparado ao reparo cirúrgico aberto. 6 ^-^ 11

 Em virtude da escassez de dados na literatura nacional a respeito da evolução comparativa entre as duas técnicas empregadas no manejo do AAA, o presente estudo tem como objetivo analisar os principais fatores de risco associados e as sobrevidas em curto (até 30 dias) e médio prazo (até 1 ano) dos pacientes submetidos à correção aberta e endovascular em hospital quaternário. O estudo foi aprovado pelo Comitê de Ética em Pesquisa da instituição sob o número 2.069.326. 

## MÉTODOS

 Este estudo retrospectivo foi realizado por meio de análise de prontuários de 45 pacientes submetidos a correção aberta ou endovascular de AAA infrarrenal no período de março de 2013 a março de 2017, em hospital quaternário. Não houve randomização formal dos pacientes tratados no serviço para escolha do método, aberto ou endovascular. Entretanto, a decisão da técnica foi tomada em conjunto com os membros da equipe, após análise da anatomia tomográfica, das comorbidades e do risco cirúrgico. Os pacientes eletivos com anatomia favorável (colo proximal > 25 mm de extensão ou ângulo < 60º e artérias ilíacas externas com diâmetro > 7 mm) ou aqueles com anatomia limítrofe (colo proximal entre 15 e 25 mm de extensão ou ângulo entre 60º e 70º), mas com risco cirúrgico elevado foram submetidos à técnica minimamente invasiva. Os demais casos eletivos foram submetidos à cirurgia aberta. Os casos urgentes (aneurismas rotos ou em expansão aguda) tiveram a indicação da técnica baseada na estabilidade do quadro clínico, anatomia favorável e disponibilidade imediata da endoprótese. Cada paciente estudado foi submetido a somente um procedimento de correção de aneurisma. Foram pesquisados 10 dados clínicos pré-operatórios e oito dados clínicos pós-operatórios, que foram inseridos em planilha eletrônica. Quatro prontuários de pacientes cirúrgico estavam incompletos (dois não possuíam dados de tempo de cirurgia, um não possuía exames laboratoriais completos disponíveis e um não possuía exame de imagem acessível para análise do diâmetro do aneurisma) e foram excluídos do estudo, totalizando 41 prontuários para serem analisados. A correlação entre as características anatômicas dos aneurismas tratados e sua evolução não foi estudada devido à indisponibilidade de exames completos para reconstrução em todos os casos. 

 A hipertensão arterial sistêmica foi definida como pressões maiores que 140 x 90 mmHg ou uso contínuo de anti-hipertensivos; diabetes melito como glicemia de jejum > 106 mg/dL ou uso de hipoglicemiantes; tabagismo como uso prévio ou atual de tabaco ou derivados; insuficiência renal como *clearance* de creatinina < 60 mL/min ou creatinina sérica > 1,5 mg/dL; e doença arterial obstrutiva periférica como índice tornozelo-braquial < 0,9 ou sinais clínicos evidentes de oclusão arterial. Outros parâmetros pesquisados foram história de infarto agudo do miocárdio inferior a 6 meses, acidente vascular cerebral, angina, dor abdominal e diâmetro do aneurisma. O AAA roto foi diagnosticado por meio de exame de imagem (ultrassom ou tomografia computadorizada). As características de evolução após a correção do AAA pesquisadas foram mortalidade hospitalar (ocorrida durante o procedimento cirúrgico ou no pós-operatório imediato), mortalidade geral (morte por qualquer causa, fora de ambiente hospitalar, após a alta), tempo em unidade de terapia intensiva menor que 24 horas, necessidade de transfusão sanguínea no intra e pós-operatório, insuficiência renal aguda (aumento de 0,5 mg/dL ou aumento em 25% no valor basal), tempo de internação hospitalar após realização da correção do AAA e duração da cirurgia. O tempo de seguimento proposto para o cálculo de sobrevida foi de até 360 dias. 

 A estatística descritiva foi obtida por média e desvio padrão. A análise inferencial intergrupos (cirurgia aberta e endovascular) foi realizada pelos testes *t* de Student para amostras independentes, Mann-Whitney ou exato de Fisher. A análise de sobrevida foi obtida pela curva de Kaplan-Meier com teste de *log-rank* para comparação entre os grupos. Foi utilizado o *software* Graphpad Prism, versão 7.0c, com intervalo de confiança (IC) de 95% e significância estatística com p < 0,05. 

## RESULTADOS

 Do total de 41 pacientes submetidos ao tratamento do AAA, 29 fizeram correção endovascular e 12 fizeram correção aberta. A maioria era do sexo masculino (n = 29; 70,7%) e a média de idade dos pacientes foi de 71 anos (mín. 56, máx. 90 anos). Do total dos procedimentos realizados, 14 pacientes foram a óbito no período de estudo. Nove casos (22%) eram AAA rotos, sendo que dois de quatro pacientes sobreviveram após o procedimento endovascular, enquanto quatro de cinco pacientes submetidos à cirurgia aberta faleceram no pós-operatório imediato. Até o período estudado, somente um caso de cirurgia aberta e nove de tratamento endovascular ainda mantinham acompanhamento ambulatorial. 

 A Tabela 1 mostra os diferentes fatores de risco presentes em ambos os grupos de pacientes tratados. Não foi possível observar diferença estatística entre os grupos. A Tabela 2 mostra a comparação entre as características pós-operatórias presentes nos grupos de pacientes submetidos à cirurgia aberta e endovascular. Foi observado que mortalidade hospitalar geral, tempo de internação e tempo de cirurgia foram estatisticamente menores no grupo submetido ao tratamento endovascular. O tempo de internação analisado englobou somente os pacientes que efetivamente receberam alta hospitalar, sendo que a mediana foi de 4 dias para a cirurgia aberta (mín. 3, máx. 6 dias) e de 2 dias para o tratamento endovascular (mín. 1, máx. 10 dias). 

**Tabela 1:** Fatores de risco pré-operatórios observados nos grupos de cirurgia aberta ou endovascular.

**Fatores de risco**	**Aberto**		**Endovascular**		**p**
	**n**	**%**	**n**	**%**	
Sexo					
Masculino	8	67	23	79	0,44
Feminino	4	33	6	21	
Idade (anos)	69	7,2 (DP)	72	9,5 (DP)	0,32
HAS	10	83	23	79	0,99
Diabetes melito	1	8,3	6	20,7	0,65
Tabagismo	10	83	6	55	0,15
IRC	1	8,3	7	24	0,40
IAM < 6 m	0	0	2	8,3	0,54
Angina	2	17	2	6,9	0,56
AVC	0	0	1	3,6	0,99
DAOP	1	8,3	5	18	0,64
Dor abdominal	7	58	13	44	0,50
AAA roto	5	42	4	14	0,09
Diâmetro aneurisma (cm)	6,8	2,3 (DP)	6,1	1,7 (DP)	0,30

AAA, aneurisma de aorta abdominal; AVC, acidente vascular cerebral; DAOP, doença arterial obstrutiva periférica; DP, desvio padrão; HAS, hipertensão arterial sistêmica; IAM, infarto agudo do miocárdio; IRC, insuficiência renal crônica.

**Tabela 2:** Comparação das variáveis pós-operatórias entre os grupos tratados (cirurgia aberta e endovascular).

**Evolução**	**Aberto**		**Endovascular**		**p**
	**n**	**%**	**n**	**%**	
Mortalidade hospitalar	7	58	4	14	0,006
Mortalidade geral (em 1 ano) ^*^	7	58	7	24	0,06
Mortalidade hospitalar (eletivos)	2	29	2	8	0,18
UTI < 24 h	1	6,7	5	31	0,17
Transfusão sanguínea	6	60	10	34	0,26
IRA pós-procedimento	2	20	6	21	0,99
Tempo de internação (dias) ^*^	4,4	1,1 (DP)	3,0	1,9 (DP)	0,02
Tempo de cirurgia (min)	163	36 (DP)	115	46 (DP)	0,005

DP, desvio padrão; IRA, insuficiência renal aguda; UTI, unidade de terapia intensiva;

* Excluindo os óbitos intra-hospitalares.

 A sobrevida dos pacientes tratados foi analisada de forma global (agrupando todos os casos eletivos e urgentes) e somente com os casos eletivos, tanto no curto (até 30 dias) quanto no médio prazo (até 1 ano), para ambos os tipos de tratamento, aberto ou endovascular ( Figura 1 ). Foi observada uma diferença significativa na sobrevida global dos pacientes tratados por via endovascular, independentemente do tempo de acompanhamento. Entretanto, quando analisados somente os casos eletivos, não houve diferença na sobrevida em curto ou médio prazo. A sobrevida dos casos eletivos em 30 dias foi de 71% na cirurgia aberta e 76% na endovascular (p = 0,44), enquanto em 360 dias ambas foram de 71% (p = 0,34), respectivamente. Na análise de todos os pacientes tratados, a sobrevida em 30 dias foi de 37% para o reparo aberto e 72% para o endovascular (p = 0,01), enquanto em 360 dias foi de 37% e 67%, respectivamente (p = 0,01). 

**Figura 1:** Curvas de sobrevida de Kaplan-Meier com teste *log-rank* para os pacientes tratados. Em a e b, a curva de sobrevida global para todos os pacientes (eletivos e urgentes), para 30 e 360 dias, evidencia diferença significativa entre os grupos. Em c e d, a análise isolada dos pacientes eletivos não aponta diferença de sobrevida relacionada à técnica de tratamento empregada.

## DISCUSSÃO

 Apesar de a correção aberta do AAA ter sido iniciada em 1951 com Dubost e ter se mantido como terapia padrão ao longo dos anos, o reparo endovascular foi realizado com êxito somente em 1990 por Parodi et al. 12 Desde então, tem sido alternativa ao reparo cirúrgico aberto. O procedimento endovascular foi desenvolvido com a finalidade de oferecer um tratamento menos traumático para correção de aneurismas, como uma opção para idosos, pacientes de alto risco e com doenças associadas que interfeririam no risco do procedimento convencional. Apresenta uma alta taxa de sucesso e menores complicações perioperatórias em comparação ao reparo cirúrgico aberto. Suas complicações geralmente estão relacionadas a algum aspecto técnico, como dificuldade no acesso vascular e na colocação da prótese, integridade estrutural, migração da prótese e *endoleaks* (vazamentos). Entretanto, a conversão para o reparo cirúrgico aberto é rara e, quando ocorrem, as complicações tardias podem ser tratadas por técnicas endovasculares. 13 ^,^ 14 Atualmente, a indicação preferencial é pela técnica minimamente invasiva, sendo a cirurgia aberta restrita àqueles pacientes sem condições anatômicas para o implante de endoprótese ou aos portadores de AAA roto instáveis. Isso corrobora os nossos achados de inexistência de diferença estatística entre as características epidemiológicas entre os dois grupos. 

 Mesmo quando submetidos ao reparo cirúrgico aberto, os pacientes com AAA roto apresentam mortalidade de aproximadamente 50%, taxa esta que não tem se alterado ao longo dos últimos anos. 15 Pacientes com AAA cujo diâmetro transverso máximo mede entre 5,5 e 5,9 cm apresentam taxa anual de ruptura de 9,4%, que pode chegar a 32,5% quando o aneurisma atinge 7 cm. 16 No entanto, o reparo cirúrgico aberto envolve recuperação prolongada e mortalidade perioperatória não desprezível, 17 com taxas variáveis, apresentando valor médio de 3,1% no Reino Unido. 18 Entre alguns autores brasileiros, encontramos resultados semelhantes com taxas de 3,3 a 5,3%. 19 ^-^ 21 Porém, dependendo da configuração anatômica da aorta e das artérias de acesso, em caso de colo proximal mais curto, tortuoso e dilatado, bem como também vasos ilíacos tortuosos e estreitos, há a necessidade de correção aberta. 

 Em um estudo brasileiro retrospectivo de pacientes que foram submetidos ao tratamento endovascular entre junho de 1996 e fevereiro de 2004, com base na análise de banco de dados do projeto *European Collaborators on Stent-graft Techniques for Abdominal Aortic Aneurysm Repair* (EUROSTAR), mostrou-se uma duração média do procedimento de 137 minutos (25 a 287 min) e internação hospitalar média de 6 dias (0 a 163). 22 No presente estudo, foi possível observar uma mediana do tempo de internação menor no tratamento endovascular (2 dias) em comparação à cirurgia aberta (4 dias), ambas menores que no estudo acima citado. Isso pode ser indicativo de melhoria das técnicas, materiais e consolidação da organização da equipe cirúrgica. O tempo médio de procedimento endovascular também foi menor (115 min), acompanhando o mesmo raciocínio. No estudo EVAR-1, 23 mais recentemente, a duração do tratamento endovascular também não foi superior à correção aberta, o que concorda com o que foi observado no nosso estudo. 

 Após a realização do EVAR-1, 23 ficou comprovada a redução da mortalidade perioperatória (4,7% para o reparo aberto *vs.* 1,7% para o endovascular). Em outro estudo controlado e randomizado, a taxa foi menor com a técnica endovascular (1,2 *vs.* 4,6%). 24 Em nosso meio, Mendonça et al. 25 compararam o tratamento aberto com o endovascular para o AAA com anatomia favorável. A mortalidade foi de 6,45% para o tratamento aberto e de 5,55% para o tratamento endovascular, não havendo uma diferença estatisticamente significativa. No presente trabalho, não observamos diferença na mortalidade intra-hospitalar entre os casos eletivos aberto e endovascular (29 *vs.* 8%, respectivamente; p = 0,18), assim como não observamos diferença na mortalidade em 30 dias (71 *vs.* 76%; p = 0,44). 

 Goshima et al. 26 afirmam que o resultado padrão para reparação aberta deve ser de 3,1% e, em seu estudo, a mortalidade do tratamento endovascular foi nula. Mas os mesmos autores, ao apresentar os casos complexos, relacionam uma mortalidade hospitalar de 14,1%, semelhante ao nosso estudo, que incluiu pacientes com aneurisma roto. No estudo EVAR-2, 27 ^,^ 28 no qual os pacientes inadequados para o reparo aberto foram randomizados para o tratamento endovascular ou acompanhamento clínico, a mortalidade de 30 dias do grupo operado foi de 9% (5-15%; IC95%). Isso reforça a ideia de que casos mais complexos, mesmo quando submetidos ao tratamento endovascular, podem apresentar taxa de mortalidade mais elevada. Em nosso estudo, nos confrontamos com uma situação semelhante, de alta taxa de mortalidade em 30 dias mesmo nos casos eletivos e por via endovascular. Além de considerarmos que tratamos pacientes com alto risco cirúrgico, oriundos de outros municípios e sem controle adequado dos fatores de risco, o fato de se tratar de hospital de ensino, com curva de aprendizado multiprofissional para o tratamento da doença aórtica, certamente gera um viés que não pode ser menosprezado. 

 O estudo multicêntrico holandês DREAM 24 também demonstrou tendência de menor mortalidade operatória (até 30 dias) no tratamento endovascular quando comparado à cirurgia aberta. Entretanto, no nosso estudo, foi observado que pacientes de ambos os grupos, tratados eletivamente, não apresentaram diferença na sobrevida em 30 dias ou 360 dias. A escolha não aleatorizada da técnica a ser empregada deve ter tido papel fundamental na sobrevida semelhante entre os grupos. 

 No entanto, foi possível observar que no seguimento de 2 e 4 anos do DREAM e do EVAR-1, foi demonstrada mortalidade semelhante nos dois grupos em longo prazo. 27 ^,^ 29 No nosso estudo, houve diferença entre os dois grupos na sobrevida geral (rotos + eletivos) nos primeiros 30 dias, mas em médio prazo os resultados se igualam, compatível com o estudo acima citado. 

 Entre os pontos que podem ser considerados negativos do presente estudo retrospectivo está o número pequeno de casos, a perda de acompanhamento de pacientes que moram em outras microrregiões e a ausência de informações sobre fatores de risco em alguns prontuários analisados. Para uma melhor compreensão do assunto abordado neste trabalho, faz-se necessário realizar estudos com maior número de pacientes, de preferência por meio de colaboração multicêntrica, estabelecer protocolos prospectivos de acompanhamento e seguir em longo prazo (5 anos). 

## CONCLUSÃO

 Observamos que pacientes submetidos ao tratamento endovascular eletivo apresentaram sobrevida em curto e médio prazo semelhante àqueles submetidos à cirurgia aberta eletiva. Tempo de internação, tempo de cirurgia e mortalidade hospitalar foram menores no grupo endovascular. Não houve diferença nas características epidemiológicas e na presença de fatores de risco entre os pacientes submetidos a ambas as técnicas, sendo os critérios anatômicos e o julgamento do cirurgião utilizados para definir o tratamento a ser instituído.
